# Reducing anastomotic leakage in TaTME by mucosal coverage of staple lines: a pilot study with preliminary results

**DOI:** 10.1186/s12893-023-02071-x

**Published:** 2023-06-10

**Authors:** Katsuya Deguchi, Yasumitsu Hirano, Naoto Okazaki

**Affiliations:** Department of Gastroenterological Surgery, Saitama Medical University International Medical Center, 1397-1 Yamane, Hidaka, 350-1298 Saitama Japan

**Keywords:** Mucosal coverage, Staple line, TaTME, Anastomotic leakage, Rectal cancer

## Abstract

**Purpose:**

We have performed a single stapled anastomosis with double purse-string sutures as a Trans anal Total Mesorectal Excision (TaTME) reconstruction for low rectal cancer. We report an attempt to control local infection and reduce anastomotic leakage (AL) at this anastomotic site.

**Patients and methods:**

Fifty-one patients who underwent TaTME for low rectal cancer from April 2021 to October 2022 were included. TaTME was performed by two teams, and reconstruction was performed by anastomosis with a single stapling technique (SST). After the anastomosis was thoroughly cleaned, Z sutures were placed parallel to the staple line to suture the mucosa on the oral and anal side of the staple line and to cover the staple line circumferentially. Data on operative time, Distal Margin (DM), recurrence and postoperative complications including AL were prospectively collected.

**Results:**

The mean age of patients was 67 years. There were 36 males and 15 females. The overall mean operative time was 283.1 min, and the mean Distal Margin was 2.2 cm. Postoperative complications were observed in 5.9% of the patients, but no AL was observed, nor any serious complications with Clavien-Dindo ≥ 3 grade. Of the 49 cases excluding Stage 4, postoperative recurrence was observed in 2 cases (4.9%).

**Conclusion:**

In patients with lower rectal cancer who underwent TaTME, additional mucosal coverage of the anastomotic staple line by transanal manipulation after reconstruction may be associated with a reduction in the incidence of postoperative AL. Further studies including late anastomotic complications are needed.

## Introduction

Although endoscopic surgery has steadily penetrated the field of colorectal cancer surgery, the difficulty of endoscopic surgery for rectal cancer is still high, and there are certain concerns about its curative potential. In recent years, Trans anal Total Mesorectal Excision (TaTME) has been attracting attention along with robotic surgery to solve such procedural problems, and is recognized worldwide.

Acute leakage (AL) is probably the most serious complication of rectal cancer surgery, associated with increased postoperative mortality and with long-term consequences, including adverse functional and oncologic outcomes. increased costs associated with treatment of complications and prolonged ICU stays. Although there have been many previous studies on the causes of suture failure, Ferko A et al.‘s analysis of photographic documentation of endoscopic findings of postoperative anastomosis of rectal cancer suggests an association between local ischemia of the anastomotic staples and local infection and AL [1].

In our institution, TaTME for low rectal cancer has been introduced in April 2021, and the reconstructive method is a single-staple anastomosis with double purse-string suture. After stapling, the low stapling line can be clearly recognized from the anorectal side, and additional procedures can be performed under direct vision with hand-stitched sutures. We report our attempt to control local infection at the anastomosis and to reduce AL.

## Patients and methods

Patients who underwent TaTME for low rectal cancer from April 2021 to October 2022 at Saitama Medical University International Medical Center were included. Data were obtained from prospectively collected databases and electronic medical records. Preoperative chemoradiation therapy (CRT) was routinely administered to patients diagnosed as T3 or deeper on preoperative examination. Lateral pelvic lymph node dissection was performed in patients with clinically suspected lymph node metastases on preoperative examination. Preoperative bowel preparation consisted of mechanical pretreatment and oral antimicrobials administered two days prior to surgery.

Data on operative time, postoperative distal margin, and postoperative complications including suture failure were prospectively collected.

The diagnosis of anastomotic leakage is confirmed by abdominal CT when there are clinical findings such as elevated white blood cell counts, inflammatory findings, or other fever, except in the acute postoperative period. The diagnosis is also made based on normal changes in drainage from a drain inserted dorsally to the anastomosis until 5 POD postoperatively. Acute AL was defined as suture failure occurring within 30 days postoperatively and was evaluated with respect to Clavien-Dindo1 or greater.

### Surgical technique

TaTME was performed using a two-team hybrid technique, with the abdominal and transanal operations performed simultaneously. Abdominal manipulation is standardized in our hospital and was performed with 5 ports. The medial approach with ligation of the inferior mesenteric artery and vein was performed first, and then the lateral approach was performed. The splenic flexure was only performed when tension was placed on the anastomosis.

For transanal manipulation, a GelPOINT ® Path (Applied Medical, Rancho Santa Margarita, CA, USA) was applied to the anus and then the mucosa was marked 2 cm anorectally from the tumor. The anal side of the tumor is closed with a purse-string suture near the midpoint between the marking and the tumor to prevent tumor dissemination. The rectal wall is dissected in all layers, and the anterior wall is dissected along the prostate or vagina to the perineal translocation. The posterior wall was dissected by selecting an exfoliation layer according to the progression of the cancer.

After the specimen was removed from the body through the umbilicus, the proximal resection line was determined after evaluation of blood flow with ICG, and the oral bowel was dissected. Unlike conventional low anterior resection, the anorectal end was open at the time of reconstruction, so it was closed with a purse string suture and anastomosed with a single stapling technique (SST) using an automated anastomosis device.

**Fig. 1:**
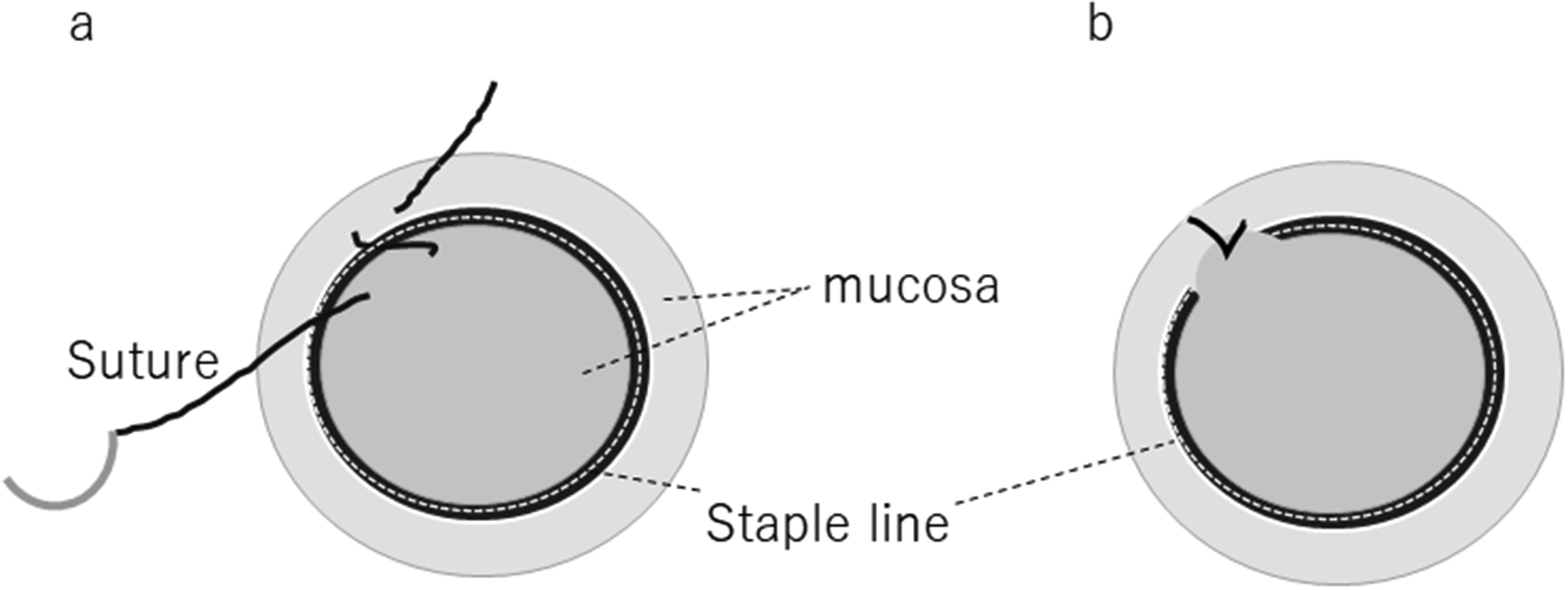
**a:** Mucocutaneous suture on the oral and anal side of the staple line by applying a Z-suture parallel to the staple line. **b:** The Z suture partially covers the staple line

**Fig. 2:**
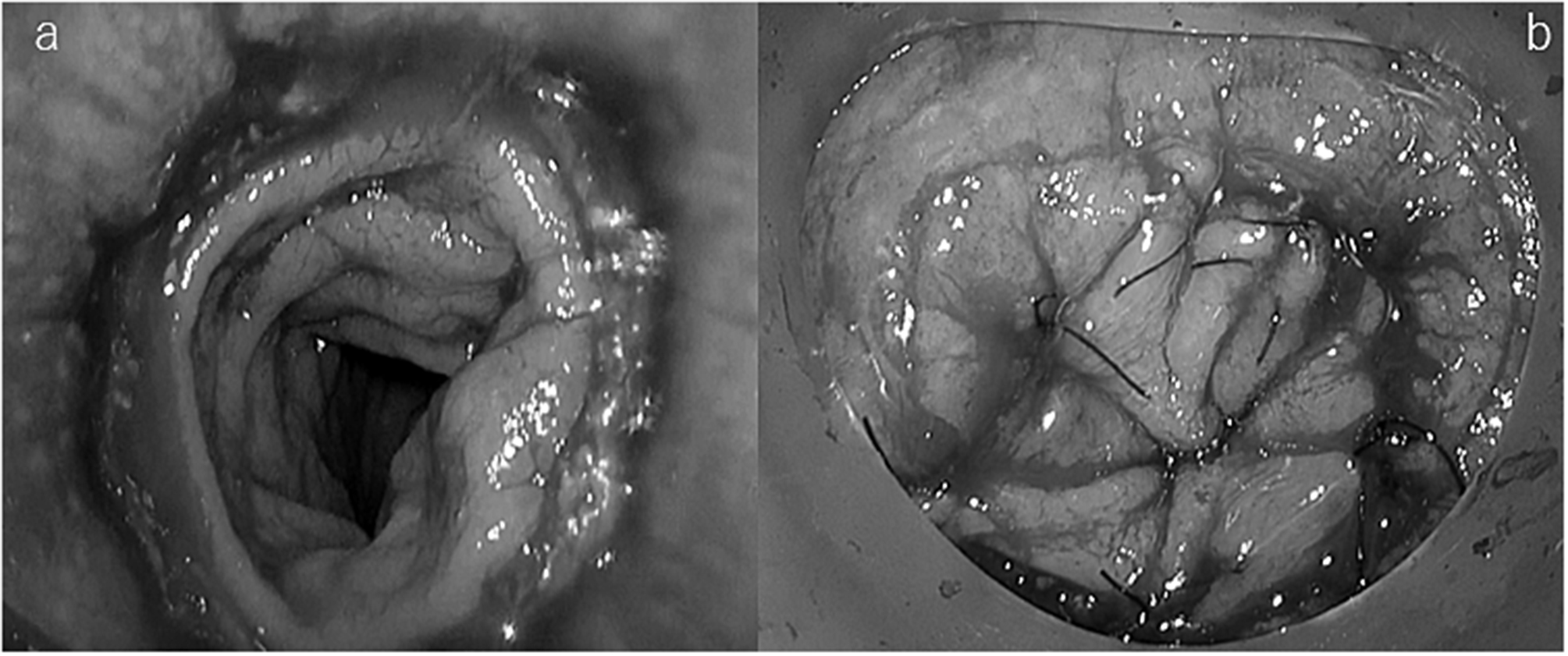
**a:** Suture line before additional sutures. **b:** Suture LINE after additional suture is fully circumferentially coated

The transanal procedure was performed using a Lone Star retractor (Cooper Surgical, Inc., USA) with the anastomosis fully pulled out anally. The stapler anastomosis was thoroughly checked for completeness, blood supply to the colonic mucosa, and signs of tension-free anastomosis, and staples and perpendicular sutures were placed for physical reinforcement purposes at the anastomosis in those cases where necessary. The anastomosis was then thoroughly washed with isodine saline and a Z suture was applied parallel to the staple line using PDS II 4/0 sutures (polydiaxonone, Ethicon, Johnson & Johnson, USA) to suture the mucosa on the oral and anal side of the staple line and the staple line was then covered circumferentially (Figs. 1 and 2). The operation was terminated by inserting a drainage tube transanally.

## Results

The mean age of the patients was 67 years. There were 36/51 (70.6%) males and 15/51 (29.4%) females, and neoadjuvant therapy was indicated in 22/51 (43.1%) of the group. Diversion ileostomy or colostomy was performed in 46 (90.2%) patients. Lateral pelvic lymph node dissection was performed in 11 patients (21.6%).

The overall mean operative time was 283.1 min, and the mean Distal Margin was 2.2 cm, with no patient having a Distal Margin of less than 0.5 cm. No patient died postoperatively, and postoperative complications were observed in 5.9% (3/51) of the patients (2 cases of ileus, 1 case of milk ascite), but no early AL was observed, nor any serious complications with Clavien-Dindo ≥ 3 grade. Of the 49 cases excluding Stage 4, postoperative recurrence was observed in 2 cases (4.9%). One case each was in a lateral pelvic lymph node and lung metastasis (Table 1).

**Table 1:** Patient demographics and outcomes

Median age, years (range)	68.0 (46–83)
Sex (male/female)	(36/15)
Median distance from anal verge, cm (range)	5.43(3–11)
Neoadjuvant therapy	22 (43.1%)
Chemoradiotherapy	18
Chemotherapy	2
Total neoadjuvant therapy	2
Diversion ileostomy or colostomy (%)	46 (90.2)
Lateral pelvic lymph node dissection (%)	11 (21.6)
Total operative time, min (range)	283.1 (144–439)
Estimated blood loss, mL (range)	84.6 (10–450)
Distal margin, cm (range)	2.2 (0.5–5.7)
Postoperative complication (%)	3 (5.9)
Clavien-Dindo ≥ 3	0
Anastomotic leakage	0
Mortality	0
Recurrence (Stage I-III) (%)	2 (4.1%)

## Discussion

TaTME was first reported worldwide in 2013 by Lacy et al. [2]. Subsequently, in 2014, Denost et al. reported that trans-anal TME had a lower CRM-positive rate than trans-abdominal TME in a randomized controlled trial [3]. In a prospective international registry study published in 2017, the R0 resection rate was 97.3% and the CRM-positive rate was 2.4%, indicating the favorable results of taTME [4]. However, a 2019 report from the same registry showed early suture failure in 7.8% of cases, late suture failure in 2.0% of cases, and anastomosis-related complications in 15.7% of cases, suggesting the need for further technical innovation [5].

In general, suture failure is a complication requiring attention in rectal cancer surgery, and it is extremely important to search for factors associated with suture failure in order to prevent its development. In general, suture failure has been considered to be caused by systemic factors such as low nutrition, vitamin C deficiency, calcium deficiency, coexistence of chronic diseases such as diabetes, prolonged administration of steroids and antibiotics, and local factors such as anastomotic regional blood flow, edema, and degree of tension in the anastomotic region.

Recently, it has been hypothesized that AL results from infectious complications caused by local enteric bacteria and that healing at the anastomosis is impaired by a local increase in collagenase activity [6], and Freko A et al. reported that anastomotic healing disorders and “restlessness” observed in their analysis of photographic documentation of endoscopic findings of anastomosis in rectal cancer surgery are associated with local ischemia and local infection, and they reported that the anastomotic healing disorders and restlessness in the anastomotic site are associated with local ischemia and local infection in a study of double-stapled anterior rectal cancer after lower anterior resection of the rectum [1]. The authors reported that the combination of double-staple reinforcement with a unique circular mucosal suture and povidone-iodine-impregnated vacuum sponge drainage resulted in a low rate of early suture failure in patients undergoing reconstruction using the double-stapled technique after low anterior resection of rectal cancer [7].

As indicated by the 7.8% early suture failure rate and 2.0% late suture failure rate reported in the aforementioned prospective international registry study [5], it is not surprising that the anastomosis is further anorectal than DST and the anastomosis method is SST in TaTME Enomoto H et al. established a method of suturing perpendicular to the staple for physical reinforcement of the anastomosis after anastomosis after TaTME and reported early suture failure in 1.8% and late suture failure in 5.5% of cases [8].

Based on the hypothesis that inflammation at the staple line, in addition to physical anastomotic weakness, could be the cause of suture failure, we performed chemical bowel pretreatment preoperatively, and after anastomosis, with the aim of reducing local inflammation caused by intestinal bacteria at the staple line of the anastomosis, we thoroughly cleaned the anastomotic site and After the anastomosis, the staple line is thoroughly cleaned, and then a Z-anastomosis is applied parallel to the staple line to cover the staple line by pulling the mucosa on the mouth and anus sides of the staple line. We have performed this method in 51 cases to date, and have not observed a single case of early AL, and have obtained extremely good results with no delayed anastomotic problems in 22 cases in which the anus has been closed to date.

Limitations of this study is that we have not been able to monitor the outcome of late suture failure. In addition, our pilot results suggest the possibility of preventing acute leaks by various attempts, such as preoperative oral antibiotics and mechanical bowel preparation, ICG evaluation of the oral bowel, transanal anastomotic reinforcement and staple line covering, and transanal tube decompression, and therefore the value and weight of each step cannot be clearly identified. Therefore, it is not possible to clearly identify the value or weight of each individual step, but only the combined effect of the individual measures. In addition, since this technique was adopted at the same time as the start of TaTME, there is no comparison group. Before the introduction of this technique, the suture failure rate of conventional laparoscopic surgery for lower rectal cancer in our department was 14.5% (17/117). Although this is not directly comparable because of the many differences in anastomosis and other techniques, we believe that rectal resection by TaTME and additional mucosal coverage of the anastomotic staple line by transanal manipulation may be associated with a lower incidence of postoperative AL.

In patients who underwent TaTME after evaluation of intestinal blood flow on oral side by ICG and appropriate intestinal pretreatment, it was suggested that postoperative suture failure could be considerably reduced by additional mucosal coverage of the staple line in addition to anastomotic reinforcement by transanal manipulation after reconstruction. Further studies are needed to clarify the effect of each measure.

## Data Availability

The datasets generated and/or analyzed during the current study are not publicly available due to privacy or ethical restrictions but are available from the corresponding author on reasonable request.
